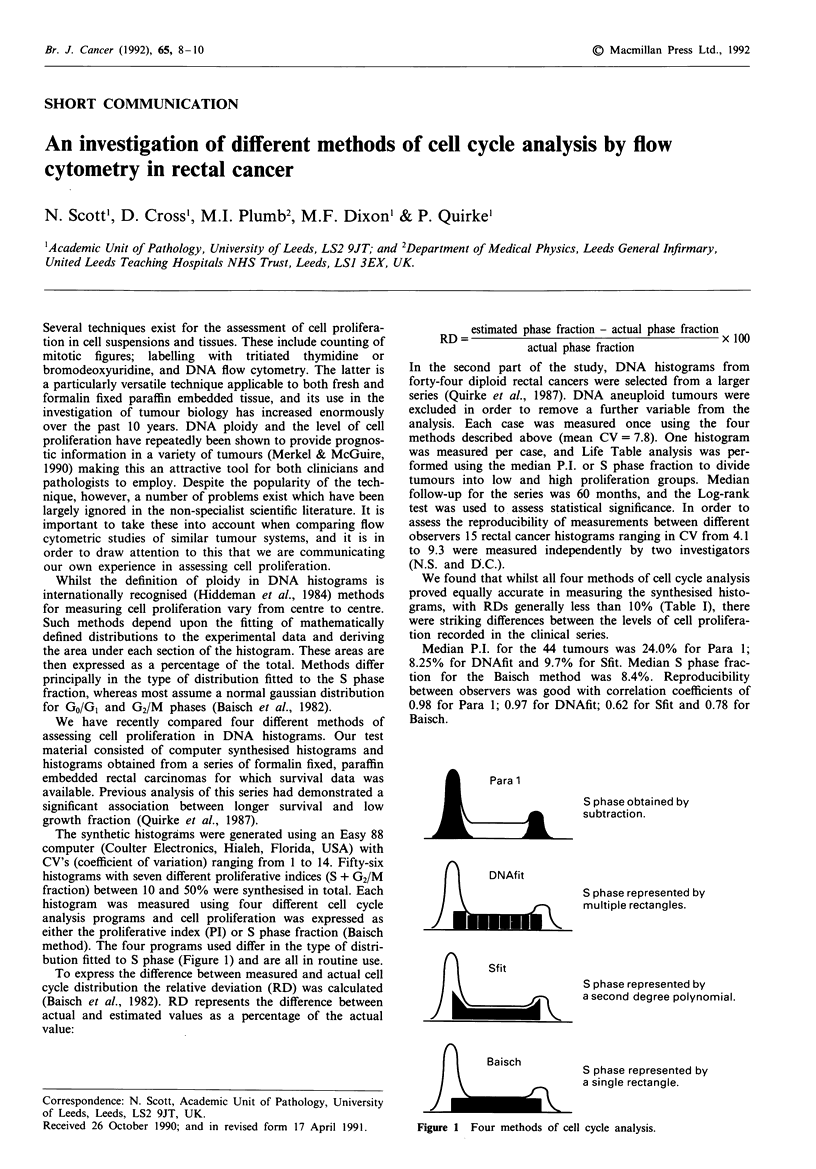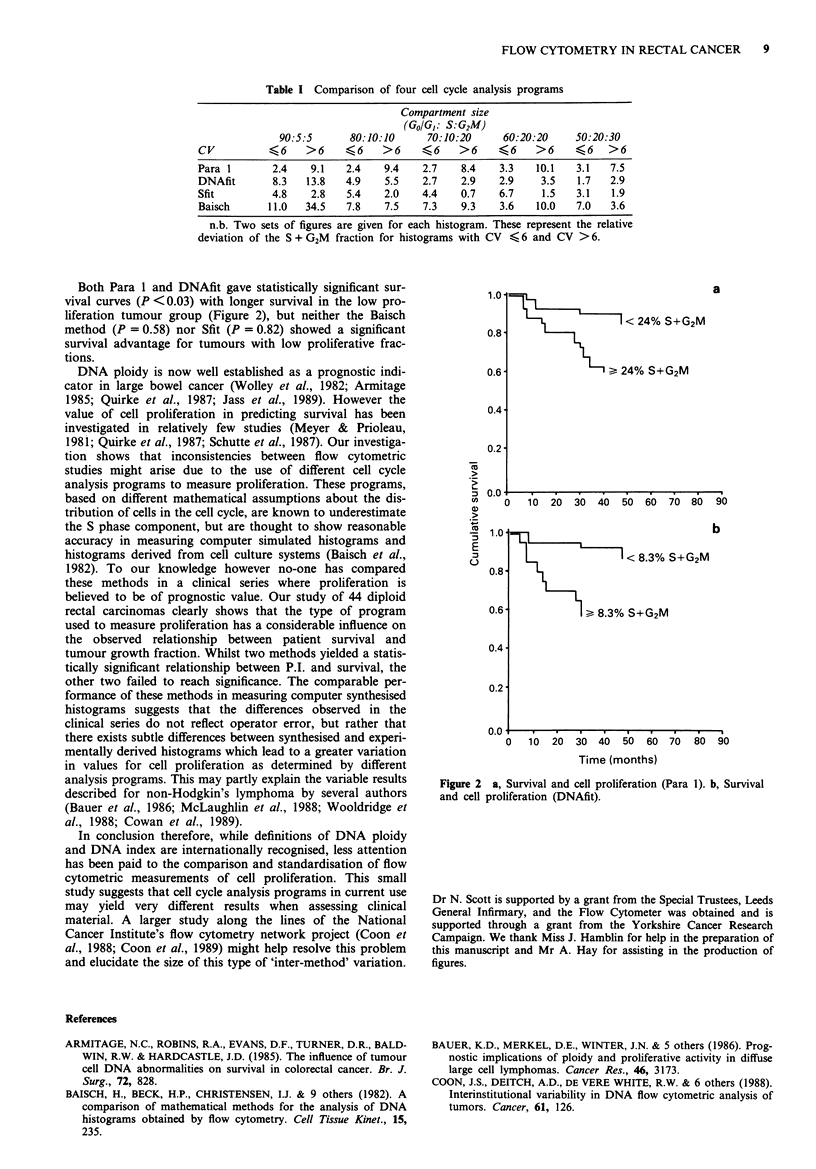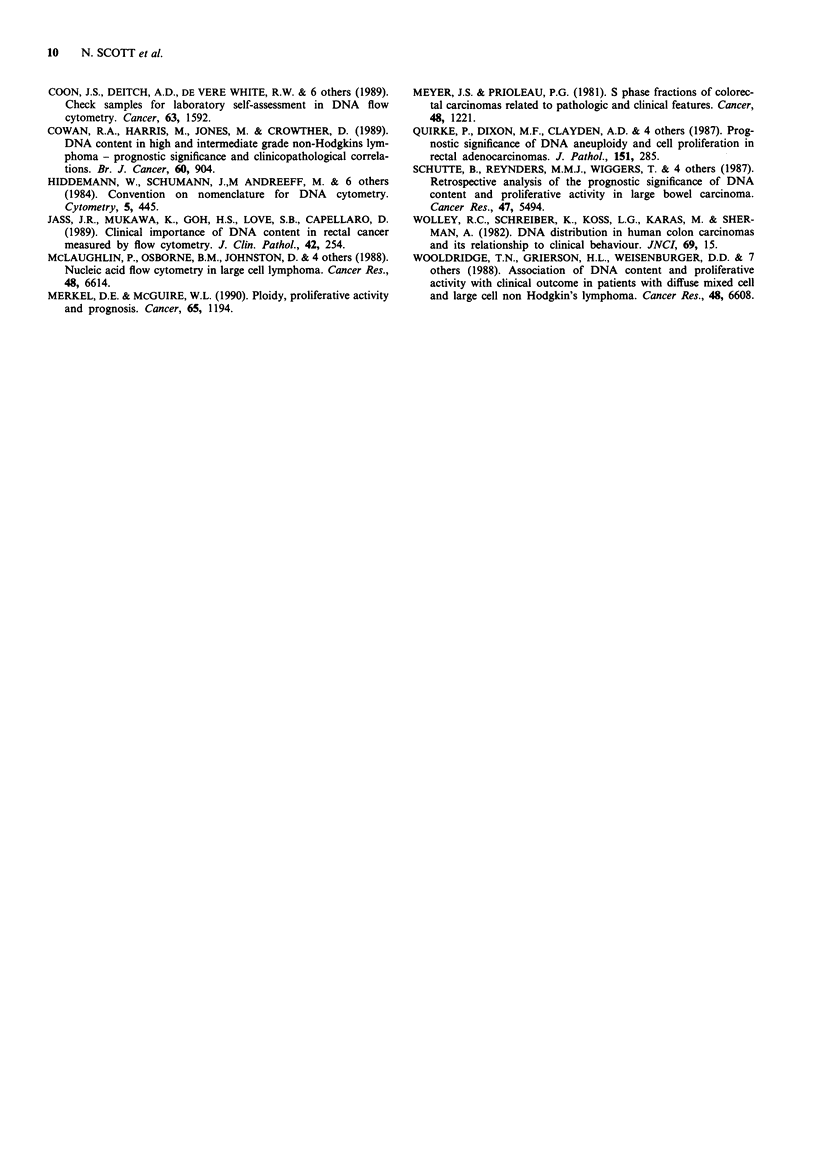# An investigation of different methods of cell cycle analysis by flow cytometry in rectal cancer.

**DOI:** 10.1038/bjc.1992.2

**Published:** 1992-01

**Authors:** N. Scott, D. Cross, M. I. Plumb, M. F. Dixon, P. Quirke

**Affiliations:** Academic Unit of Pathology, University of Leeds, UK.


Br. J. Cancer (1992), 65, 8-10 © Macmillan Press Ltd., 1992

SHORT COMMUNICATION

### An investigation of different methods of cell cycle analysis by flow cytometry in rectal cancer

N. Scott[1], D. Cross[1], M.I. Plumb[2], M.F. Dixon[1] & P. Quirke[1]

[1]*Academic Unit of Pathology, University of Leeds, LS2 9JT; and* [2]*Department of Medical Physics, Leeds General Infirmary, United Leeds Teaching Hospitals NHS Trust, Leeds, LS1 3EX, UK.*

Several techniques exist for the assessment of cell proliferation in cell suspensions and tissues. These include counting of mitotic figures; labelling with tritiated thymidine or bromodeoxyuridine, and DNA flow cytometry. The latter is a particularly versatile technique applicable to both fresh and formalin fixed paraffin embedded tissue, and its use in the investigation of tumour biology has increased enormously over the past 10 years. DNA ploidy and the level of cell proliferation have repeatedly been shown to provide prognostic information in a variety of tumours (Merkel & McGuire, 1990) making this an attractive tool for both clinicians and pathologists to employ. Despite the popularity of the technique, however, a number of problems exist which have been largely ignored in the non-specialist scientific literature. It is important to take these into account when comparing flow cytometric studies of similar tumour systems, and it is in order to draw attention to this that we are communicating our own experience in assessing cell proliferation.

Whilst the definition of ploidy in DNA histograms is internationally recognised (Hiddeman *et al.*, 1984) methods for measuring cell proliferation vary from centre to centre. Such methods depend upon the fitting of mathematically defined distributions to the experimental data and deriving the area under each section of the histogram. These areas are then expressed as a percentage of the total. Methods differ principally in the type of distribution fitted to the S phase fraction, whereas most assume a normal gaussian distribution for $G_0/G_1$ and $G_2/M$ phases (Baisch *et al.*, 1982).

We have recently compared four different methods of assessing cell proliferation in DNA histograms. Our test material consisted of computer synthesised histograms and histograms obtained from a series of formalin fixed, paraffin embedded rectal carcinomas for which survival data was available. Previous analysis of this series had demonstrated a significant association between longer survival and low growth fraction (Quirke *et al.*, 1987).

The synthetic histograms were generated using an Easy 88 computer (Coulter Electronics, Hialeh, Florida, USA) with CV's (coefficient of variation) ranging from 1 to 14. Fifty-six histograms with seven different proliferative indices ($S + G_2/M$ fraction) between 10 and 50% were synthesised in total. Each histogram was measured using four different cell cycle analysis programs and cell proliferation was expressed as either the proliferative index (PI) or S phase fraction (Baisch method). The four programs used differ in the type of distribution fitted to S phase (Figure 1) and are all in routine use.

To express the difference between measured and actual cell cycle distribution the relative deviation (RD) was calculated (Baisch *et al.*, 1982). RD represents the difference between actual and estimated values as a percentage of the actual value:

$$RD = \frac{\text{estimated phase fraction} - \text{actual phase fraction}}{\text{actual phase fraction}} \times 100$$

In the second part of the study, DNA histograms from forty-four diploid rectal cancers were selected from a larger series (Quirke *et al.*, 1987). DNA aneuploid tumours were excluded in order to remove a further variable from the analysis. Each case was measured once using the four methods described above (mean CV = 7.8). One histogram was measured per case, and Life Table analysis was performed using the median P.I. or S phase fraction to divide tumours into low and high proliferation groups. Median follow-up for the series was 60 months, and the Log-rank test was used to assess statistical significance. In order to assess the reproducibility of measurements between different observers 15 rectal cancer histograms ranging in CV from 4.1 to 9.3 were measured independently by two investigators (N.S. and D.C.).

We found that whilst all four methods of cell cycle analysis proved equally accurate in measuring the synthesised histograms, with RDs generally less than 10% (Table I), there were striking differences between the levels of cell proliferation recorded in the clinical series.

Median P.I. for the 44 tumours was 24.0% for Para 1; 8.25% for DNAfit and 9.7% for Sfit. Median S phase fraction for the Baisch method was 8.4%. Reproducibility between observers was good with correlation coefficients of 0.98 for Para 1; 0.97 for DNAfit; 0.62 for Sfit and 0.78 for Baisch.

**Figure 1** Four methods of cell cycle analysis.

Correspondence: N. Scott, Academic Unit of Pathology, University of Leeds, Leeds, LS2 9JT, UK.
Received 26 October 1990; and in revised form 17 April 1991.

**Table I** Comparison of four cell cycle analysis programs

| | Compartment size ($G_0/G_1$: $S$:$G_2M$) | | | | | | | | | |
|---|---|---|---|---|---|---|---|---|---|---|
| | *90:5:5* | | *80:10:10* | | *70:10:20* | | *60:20:20* | | *50:20:30* | |
| *CV* | $\leqslant 6$ | $>6$ | $\leqslant 6$ | $>6$ | $\leqslant 6$ | $>6$ | $\leqslant 6$ | $>6$ | $\leqslant 6$ | $>6$ |
| Para 1 | 2.4 | 9.1 | 2.4 | 9.4 | 2.7 | 8.4 | 3.3 | 10.1 | 3.1 | 7.5 |
| DNAfit | 8.3 | 13.8 | 4.9 | 5.5 | 2.7 | 2.9 | 2.9 | 3.5 | 1.7 | 2.9 |
| Sfit | 4.8 | 2.8 | 5.4 | 2.0 | 4.4 | 0.7 | 6.7 | 1.5 | 3.1 | 1.9 |
| Baisch | 11.0 | 34.5 | 7.8 | 7.5 | 7.3 | 9.3 | 3.6 | 10.0 | 7.0 | 3.6 |

n.b. Two sets of figures are given for each histogram. These represent the relative deviation of the $S + G_2M$ fraction for histograms with CV $\leqslant 6$ and CV $>6$.

Both Para 1 and DNAfit gave statistically significant survival curves ($P < 0.03$) with longer survival in the low proliferation tumour group (Figure 2), but neither the Baisch method ($P = 0.58$) nor Sfit ($P = 0.82$) showed a significant survival advantage for tumours with low proliferative fractions.

DNA ploidy is now well established as a prognostic indicator in large bowel cancer (Wolley *et al.*, 1982; Armitage 1985; Quirke *et al.*, 1987; Jass *et al.*, 1989). However the value of cell proliferation in predicting survival has been investigated in relatively few studies (Meyer & Prioleau, 1981; Quirke *et al.*, 1987; Schutte *et al.*, 1987). Our investigation shows that inconsistencies between flow cytometric studies might arise due to the use of different cell cycle analysis programs to measure proliferation. These programs, based on different mathematical assumptions about the distribution of cells in the cell cycle, are known to underestimate the S phase component, but are thought to show reasonable accuracy in measuring computer simulated histograms and histograms derived from cell culture systems (Baisch *et al.*, 1982). To our knowledge however no-one has compared these methods in a clinical series where proliferation is believed to be of prognostic value. Our study of 44 diploid rectal carcinomas clearly shows that the type of program used to measure proliferation has a considerable influence on the observed relationship between patient survival and tumour growth fraction. Whilst two methods yielded a statistically significant relationship between P.I. and survival, the other two failed to reach significance. The comparable performance of these methods in measuring computer synthesised histograms suggests that the differences observed in the clinical series do not reflect operator error, but rather that there exists subtle differences between synthesised and experimentally derived histograms which lead to a greater variation in values for cell proliferation as determined by different analysis programs. This may partly explain the variable results described for non-Hodgkin's lymphoma by several authors (Bauer *et al.*, 1986; McLaughlin *et al.*, 1988; Wooldridge *et al.*, 1988; Cowan *et al.*, 1989).

In conclusion therefore, while definitions of DNA ploidy and DNA index are internationally recognised, less attention has been paid to the comparison and standardisation of flow cytometric measurements of cell proliferation. This small study suggests that cell cycle analysis programs in current use may yield very different results when assessing clinical material. A larger study along the lines of the National Cancer Institute's flow cytometry network project (Coon *et al.*, 1988; Coon *et al.*, 1989) might help resolve this problem and elucidate the size of this type of 'inter-method' variation.

**Figure 2** **a**, Survival and cell proliferation (Para 1). **b**, Survival and cell proliferation (DNAfit).

Dr N. Scott is supported by a grant from the Special Trustees, Leeds General Infirmary, and the Flow Cytometer was obtained and is supported through a grant from the Yorkshire Cancer Research Campaign. We thank Miss J. Hamblin for help in the preparation of this manuscript and Mr A. Hay for assisting in the production of figures.

### References

ARMITAGE, N.C., ROBINS, R.A., EVANS, D.F., TURNER, D.R., BALDWIN, R.W. & HARDCASTLE, J.D. (1985). The influence of tumour cell DNA abnormalities on survival in colorectal cancer. *Br. J. Surg.*, **72**, 828.

BAISCH, H., BECK, H.P., CHRISTENSEN, I.J. & 9 others (1982). A comparison of mathematical methods for the analysis of DNA histograms obtained by flow cytometry. *Cell Tissue Kinet.*, **15**, 235.

BAUER, K.D., MERKEL, D.E., WINTER, J.N. & 5 others (1986). Prognostic implications of ploidy and proliferative activity in diffuse large cell lymphomas. *Cancer Res.*, **46**, 3173.

COON, J.S., DEITCH, A.D., DE VERE WHITE, R.W. & 6 others (1988). Interinstitutional variability in DNA flow cytometric analysis of tumors. *Cancer*, **61**, 126.

COON, J.S., DEITCH, A.D., DE VERE WHITE, R.W. & 6 others (1989). Check samples for laboratory self-assessment in DNA flow cytometry. *Cancer*, **63**, 1592.

COWAN, R.A., HARRIS, M., JONES, M. & CROWTHER, D. (1989). DNA content in high and intermediate grade non-Hodgkins lymphoma – prognostic significance and clinicopathological correlations. *Br. J. Cancer*, **60**, 904.

HIDDEMANN, W., SCHUMANN, J.,M ANDREEFF, M. & 6 others (1984). Convention on nomenclature for DNA cytometry. *Cytometry*, **5**, 445.

JASS, J.R., MUKAWA, K., GOH, H.S., LOVE, S.B., CAPELLARO, D. (1989). Clinical importance of DNA content in rectal cancer measured by flow cytometry. *J. Clin. Pathol.*, **42**, 254.

MCLAUGHLIN, P., OSBORNE, B.M., JOHNSTON, D. & 4 others (1988). Nucleic acid flow cytometry in large cell lymphoma. *Cancer Res.*, **48**, 6614.

MERKEL, D.E. & MCGUIRE, W.L. (1990). Ploidy, proliferative activity and prognosis. *Cancer*, **65**, 1194.

MEYER, J.S. & PRIOLEAU, P.G. (1981). S phase fractions of colorectal carcinomas related to pathologic and clinical features. *Cancer*, **48**, 1221.

QUIRKE, P., DIXON, M.F., CLAYDEN, A.D. & 4 others (1987). Prognostic significance of DNA aneuploidy and cell proliferation in rectal adenocarcinomas. *J. Pathol.*, **151**, 285.

SCHUTTE, B., REYNDERS, M.M.J., WIGGERS, T. & 4 others (1987). Retrospective analysis of the prognostic significance of DNA content and proliferative activity in large bowel carcinoma. *Cancer Res.*, **47**, 5494.

WOLLEY, R.C., SCHREIBER, K., KOSS, L.G., KARAS, M. & SHERMAN, A. (1982). DNA distribution in human colon carcinomas and its relationship to clinical behaviour. *JNCI*, **69**, 15.

WOOLDRIDGE, T.N., GRIERSON, H.L., WEISENBURGER, D.D. & 7 others (1988). Association of DNA content and proliferative activity with clinical outcome in patients with diffuse mixed cell and large cell non Hodgkin's lymphoma. *Cancer Res.*, **48**, 6608.